# Bactericide Activity of Cellulose Acetate/Silver Nanoparticles Asymmetric Membranes: Surfaces and Porous Structures Role

**DOI:** 10.3390/membranes13010004

**Published:** 2022-12-21

**Authors:** Ana Sofia Figueiredo, Ana Maria Ferraria, Ana Maria Botelho do Rego, Silvia Monteiro, Ricardo Santos, Miguel Minhalma, María Guadalupe Sánchez-Loredo, Rosa Lina Tovar-Tovar, Maria Norberta de Pinho

**Affiliations:** 1 CeFEMA-Center of Physics and Engineering of Advanced Materials, Instituto Superior Técnico, Universidade de Lisboa, 1049-001 Lisbon, Portugal; 2 LaPMET-Associate Laboratory of Physics for Materials and Emergent Technologies, Instituto Superior Técnico, Universidade de Lisboa, 1049-001 Lisbon, Portugal; 3 Instituto Superior de Engenharia de Lisboa, Instituto Politécnico de Lisboa, 1959-007 Lisbon, Portugal; 4 BSIRG-iBB-Institute for Bioengineering and Biosciences, Universidade de Lisboa, 1049-001 Lisbon, Portugal; 5 Associate Laboratory i4HB—Institute for Health and Bioeconomy at Instituto Superior Técnico, Universidade de Lisboa, 1049-001 Lisbon, Portugal; 6 Chemical Engineering Department, Instituto Superior Técnico, Universidade de Lisboa, 1049-001 Lisbon, Portugal; 7 Laboratório de Análises, Instituto Superior Técnico, Universidade de Lisboa, 1049-001 Lisbon, Portugal; 8 Instituto de Metalurgia, Facultad de Ingeniería, Universidad Autónoma de San Luis Potosí, San Luis Potosí 78210, Mexico

**Keywords:** cellulose acetate/silver nanocomposite ultrafiltration membranes, antimicrobial properties, polyvinylpyrrolidone-coated silver nanoparticles, surface characterization

## Abstract

The antibacterial properties of cellulose acetate/silver nanoparticles (AgNP) ultrafiltration membranes were correlated with their integral asymmetric porous structures, emphasizing the distinct features of each side of the membranes, that is, the active and porous layers surfaces. Composite membranes were prepared from casting solutions incorporating polyvinylpyrrolidone-covered AgNP using the phase inversion technique. The variation of the ratio acetone/formamide and the AgNP content resulted in a wide range of asymmetric porous structures with different hydraulic permeabilities. Comprehensive studies assessing the antibacterial activity against *Escherichia coli* (cell death and growth inhibition of bacteria in water) were performed on both membrane surfaces and in *E. coli* suspensions. The results were correlated with the surface chemical composition assessed by XPS. The silver-free membranes presented a generalized growth of *E. coli*, which is in contrast with the inhibition patterns displayed by the membranes containing AgNP. For the surface bactericide test, the growth inhibition depends on the accessibility of *E. coli* to the silver present in the membrane; as the XPS results show, the more permeable membranes (CA30 and CA34 series) have higher silver signal detected by XPS, which is correlated with a higher growth inhibition. On the other hand, the inhibition action is independent of the membrane porous structure when the membrane is deeply immersed in an *E. coli* inoculated suspension, presenting almost complete growth inhibition.

## 1. Introduction

Membrane pressure-driven processes are efficient, sustainable, and easy-to handle separation technologies even though membrane fouling still poses major drawbacks of flux reduction, modification of membrane selectivity, and reduction of membrane lifetime [1]. In particular, biofouling is originated by the deposition of microorganisms and extracellular polymeric substances (EPS) and causes high concern because microorganisms multiply over time and, even if the membrane can be regenerated, some cells remain and can multiply, re-forming a biofilm in a short time [2]. One of the most used solutions to overcome this limitation is the incorporation of nanoparticles in the polymeric structure of the membranes, which combines the properties of polymeric membranes with the characteristics of nanoparticles, intending to improve the mechanical properties of the membranes and enhance the permeation performance. Due to their known bactericidal potential, silver nanoparticles (AgNP) have been widely used to incorporate into membranes [3]. Qi et al. developed a one-pot method of synthesizing AgNP and immobilizing them by soaking a polysulfone ultrafiltration membrane in a mixture of silver nitrate, poly(ethylene glycol)methyl ether diol, and dopamine. The results indicated that membranes exhibited outstanding antibacterial properties with more than 90% of antibacterial efficiency against *Escherichia coli* and *Staphylococcus aureus* [4]. More recently, nanosilver stabilized with the polyhexamethylene biguanide hydrochloride (PHMB) was incorporated in situ onto the thin-film composite (TFC) NF90 membrane surface, and the results demonstrate that the system has a profound antibacterial effect against *Staphylococcus aureus* and *Escherichia coli* bacteria [5]. Peng et al. reported the deposition of silver nanoparticles (AgNP) on tunicate cellulose nanocrystals (TCNCs) by in situ hydrothermal reduction of silver nitrate, showing excellent antibacterial efficacy against *Staphylococcus aureus* and *Escherichia coli* [6].

In our earlier work, as an effort to overcome the biofilm phenomena, nanofiltration cellulose acetate/silver nanoparticle membranes with antimicrobial properties were developed [7].

Ultrafiltration (UF) cellulose acetate/silver nanoparticles (CA/Ag) membranes were prepared by phase inversion with polyvinylpyrrolidone-stabilized silver nanoparticles (AgNP) synthesized ex situ and incorporated in the casting solutions, with the solvent system having different acetone/formamide ratios [8]. The membranes casted with different acetone/formamide ratios displayed a range of asymmetric porous structures with tailored selective permeation properties. In the present work, the surfaces of these asymmetrical membranes were characterized by X-ray photoelectron spectroscopy (XPS), paying particular attention to the active layer to have a better understanding of the silver oxidation state and amount and its correlation with the antimicrobial effect against *Escherichia coli*. The interaction nanoparticles–bacteria and, therefore, the bactericide properties could be better explained by knowing the surface characteristics of the modified membranes. Therefore, it was aimed to shed light on the surface properties of the membranes using a surface driven technique such as XPS complemented with the analysis of the structural characteristics of the AgNP using X-ray diffraction.

Hypothesis:

The integral asymmetric porous structures of cellulose acetate membranes, some of them incorporating silver nanoparticles, are characterized by distinct features of the surfaces of the active and porous layers. The antibacterial activity against *Escherichia coli* in both silver-free and AgNP membranes can be correlated with the surface chemical composition, as well as with the silver accessibility, by detailed and systematic X-ray photoelectron spectroscopy (XPS) studies. Such analysis was never reported before.

## 2. Materials and Methods

### 2.1. Materials and Chemicals

Polyvinylpyrrolidone (PVP) (BDH Chemicals, Dubai, UAE, ~44,000 g/mol), sodium borohydride (Panreac, Barcelona, Spain, >96% purity), silver nitrate (Panreac, Barcelona, Spain, >99.8% purity), cellulose acetate (Sigma-Aldrich, Darmstadt, Germany, ~30,000 g/mol, 39.8 wt%. acetyl, corresponding to a degree of substitution of 2.5), formamide (Sigma-Aldrich, Darmstadt, Germany, ≥99.5% purity), acetone (Labchem, Zelienople, PA, USA, 99.9% purity), polyethylene glycol (PEG) (Merck-Schuchardt, Munich, Germany, —1000, 3000, 6000, 8000, 10,000 and 20,000 Da), dextran (Amersham Pharmacia Biotech AB, Staffanstorp, Sweden, 40,000 Da), nutrient broth (Becton, Dickinson and Company, Franklin Lakes, New Jersey, USA), yeast extract agar (YEA) (Biokar, Allonne, France), and *Escherichia coli* (*E. coli*) strain ATCC 700078 (WG5) were used.

### 2.2. Synthesis of Silver Nanoparticles

Silver nanoparticles were prepared and stabilized with PVP following a synthesis procedure adapted from Tashdjian et al. (2013) [9] and Figueiredo et al. (2015) [8].

### 2.3. Membranes Preparation

Cellulose acetate (CA) and cellulose acetate with AgNP (CA/Ag) flat sheet membranes were prepared by the phase inversion method [10,11].

The CA/Ag casting solutions were prepared with 0.1 and 0.4 wt% Ag, by the addition of the dispersion of AgNP in the CA casting solutions of different compositions (CA/acetone/formamide ratios used were 17/61/22, 17/53/30, and 17/49/34). The casting solutions were prepared at room temperature, according to the compositions and conditions presented in Table 1.

The CA and CA/Ag (Table 1) casting solutions were prepared according to the procedure reported by Figueiredo et al. (2015) [8]. To prepare the CA/Ag casting solutions, the AgNP dispersions were previously introduced in the acetone. The solutions were cast on a clean glass plate using a casting knife, with the gate height fixed at 0.25 mm.

### 2.4. Permeation Experiments

Permeation experiments were performed for pure water to characterize the membranes in terms of hydraulic permeability and, for organic solutes to determine the molecular weight cut-off (MWCO) of the membranes. The permeation experiments were performed in flat plate units with two detachable parts separated by a porous plate (membrane support), with a membrane surface area of 13.2 × 10^−4^ m^2^. Before the permeation experiments, the membranes were compacted for 2 h with deionized water at a transmembrane pressure of 3 bar.

#### 2.4.1. Pure Water Permeation Experiments

The hydraulic permeability (Lp) is obtained by the slope of the straight line of pure water permeate fluxes (Jp) as a function of the transmembrane pressure (ΔP). The transmembrane pressures ranged from 1 to 3 bar with a flow rate of 180 L h^−1^.

#### 2.4.2. Molecular Weight Cut-Off Experiments

The MWCO parameter is defined by the molecular weight of a given macromolecule whose rejection is higher than 91% and is obtained using the rejection coefficients of organic solutes, such as polyethylene glycol (PEG), with different molecular weight (Merck Schuchardt, Munich, Germany —1000, 3000, 6000, 8000, 10,000, and 20,000 Da) and Dextran (Amersham Pharmacia Biotech AB, Staffanstorp, Sweden —40,000 Da).

The apparent rejection coefficients (f) are defined as f = (C_f_ − C_p_)/C_f_, where C_f_ and C_p_ are the organic solute concentrations in the bulk of the feed solution and of the permeate solution, respectively. To determine the MWCO, the curve of log(f/(1−f)) is plotted as a function of the molecular weight of the organic solutes used, and the interception of this curve with the horizontal line corresponding to a rejection of 91% gives the MWCO of the membrane.

The permeation experiments were conducted using aqueous solutions of the organic solutes at 600 ppm and with a flow rate of 180 L h^−1^ at 1 bar. The organic solute concentrations in the feed and permeate solutions were determined in terms of total organic carbon (TOC) content, using a Dohrmann Total Organic Carbon Analyzer Model DC-85A.

### 2.5. X-ray Diffraction Analysis

The presence of crystalline silver in the nanoparticles was analyzed using X-ray diffraction (XRD). The characterization was performed on a dried powder sample, which was obtained by precipitation of the nanoparticles, adding acetone to the aqueous dispersion and washing with water and isopropanol. XRD analysis was carried out with a Bruker D8 Advance Diffractometer (Cu Kα radiation, λ = 1.54060 Å; 40 kV, 35 mA), using a silicon single crystal ((911) orientation) as a sample holder to minimize scattering. The powder sample was analyzed in the range of 4 to 90° 2theta, with a step size of 0.02° and a counting time of 3.35 s. To estimate the average crystallite sizes and specific crystallite size anisotropy from diffraction peak broadening reflections (using the Scherrer equation), the Rietveld refinement (Le Bail method) was performed using the Bruker software TOPAS 4.2. For each Rietveld refinement, the instrumental correction was included as determined with a standard powder sample Al_2_O_3_ (from National Institute of Standards and Technology (NIST), as standard reference material, NIST 1976a). The diffraction pattern was analyzed qualitatively using the PDF-2 (2010) of ICDD databases.

### 2.6. X-ray Photoelectron Spectroscopy

Silver nanoparticle dispersions and membrane surfaces of both active and porous layers were characterized by XPS using a XSAM800 dual anode spectrometer from KRATOS. Operating conditions, data acquisition, and data treatment were described elsewhere [12]. Binding energies were corrected from the charge shift using the binding energy of aliphatic carbon as reference (285.0 eV). The following sensitivity factors were used for quantitative purposes: 0.25 for C 1s; 0.66 for O 1s; 0.42 for N 1s; and 4.05 for Ag 3d. Membranes and the AgNP dispersions were dried in a vacuum chamber before the XPS analysis. Unlike the AgNP used for XRD analysis, the particles were not purified, and the dispersion was analyzed as prepared.

### 2.7. Membranes Bactericide Properties

The bactericide properties of the CA and CA/Ag membranes were evaluated through tests performed under different experimental conditions, where the capability of the membranes to inhibit the generalized growth of bacteria in water, specifically of *E. coli*, was tested. The specific tests to evaluate the growth inhibition of *E. coli* in the presence of the CA and CA/Ag membranes were conducted in three different ways designated by surface test, suspension test, and cell death test. The bactericide properties of the membranes were tested against *E. coli* strain ATCC 700078, also known as WG5. *E. coli* was grown in nutrient broth at 37 °C with orbital shaking (200 rpm) until the exponential growth phase was reached. Before incubation with the bacterial strain, each side of the membranes was sterilized for 30 min under ultraviolet (UV) radiation. The effectiveness of this sterilization was confirmed by incubating a sample of the sterilized membrane at 37 °C for 24 h in a Petri dish with YEA.

#### 2.7.1. Surface Test

The surfaces of the active and porous layers were tested for their antibacterial properties. A sample of the membrane was placed in a Petri dish with YEA, and 100 µL of *E. coli* suspension (2.5 × 10^5^ colony forming units (CFU)) was spread on the respective membrane surface. The plate was incubated at 37 °C for 24 h and the growth inhibition was evaluated.

#### 2.7.2. Suspension Test

A sample of the membrane was placed in a flask with 2 mL of *E. coli* suspension (2.5 × 10^5^ CFU) covering the membrane. The flask was capped and maintained at rest at room temperature for 24 h. On the next day, 10 mL of sterile water was added to the flask. The membrane was removed from the suspension and placed in a Petri dish with YEA. In parallel, the inoculated suspension was filtered through a Whatman membrane filter (0.2 µm), and this was placed in a Petri dish with YEA. Both were incubated at 37 °C for 24 h and the growth inhibition was evaluated.

#### 2.7.3. Cell Death Test

A membrane sample was immersed in a solution containing 9 mL of sterilized water and 1 mL of the exponential-growth-phase culture of *E. coli* in a nutrient broth medium (8 × 10^7^ CFU). The membrane was kept in the inoculated suspension at 37 °C with orbital shaking (200 rpm) for 18 h. To validate the bactericide effect of the membranes, samples of the suspension were collected at different contact times (143 min, 190 min, and 975 min), with the final sample taken at 18 h (1093 min) of incubation. At each time, 100 μL of the direct sample and several dilutions (10^−2^, 10^−4^, 10^−5^, and 10^−6^) were inoculated on YEA. The plates were incubated at 37 °C for 24 h, and the number of colonies was counted.

#### 2.7.4. Growth Inhibition of Bacteria in the Water

To test the membrane’s capability to inhibit the growth of bacteria in water, a membrane was immersed in water for 80 days. A sample of 10 mL of the water was filtered through a membrane filter of 0.45 µm (Whatman), the membrane filter was placed in a Petri dish with YEA and was incubated at 37 °C for 24 h. After incubation, the number of colonies was counted.

## 3. Results and Discussion

### 3.1. Membrane Synthesis and Permeation Experiments

Different asymmetric structures of cellulose acetate/AgNP (CA/Ag) membranes were synthesized with 0.1wt% Ag (CA22Ag0.1, CA30Ag0.1, and CA34Ag0.1) and 0.4wt% Ag (CA22A0.4, CA30Ag0.4, and CA34Ag0.4). Cellulose acetate (CA) membranes (CA22, CA30, and CA34) were also prepared to be used as references.

The pure water fluxes and the characteristic parameters of hydraulic permeability and MWCO of the synthesized membranes are listed in Table 2.

The addition of 0.1% wt Ag introduced an increase of hydraulic permeability values in all the prepared membranes (CA22Ag0.1, CA30Ag0.1, and CA34Ag0.1), and the incorporation of 0.4% wt Ag only enhanced the permeability of the CA22 and CA30. On the other hand, the two membranes with 0.4% wt Ag, CA30Ag0.4 and CA34Ag0.4, presented lower hydraulic permeability values when compared with the CA30Ag0.1 and CA34Ag0.1 membranes. It was observed that for the tighter membranes (CA22), the hydraulic permeability was enhanced with the incorporation of silver nanoparticles in both concentrations; for the CA30 membranes, an increase of permeability was observed with the addition of 0.1% wt Ag, but the incorporation of 0.4% wt Ag induced a decrease in the hydraulic permeability value when compared with the CA30Ag0.1. The hydraulic permeability value for the more porous structure (CA34) slightly increased (5%) with the incorporation of 0.1% wt Ag and presented a strong decrease after the addition of the higher content of AgNPs (CA34Ag0.4), the results obtained being even lower than for the CA34 (with no AgNP). Taurozzi et al. (2008) [13] observed only an increase in permeability after silver nanoparticles incorporation for the denser polysulfone membrane. A higher silver nanoparticles content in casting solutions enhanced the hydraulic permeability of the denser CA22 membranes; however, for the CA30 and CA34 membranes, the increase of silver content from 0.1 to 0.4%Ag led to a decrease of the hydraulic permeability, which may be due to the pore-blocking by the AgNP [14] as a result of aggregation due to a lower compatibility between the silver dispersion and the casting solutions.

### 3.2. XRD Results

**Figure 1:**
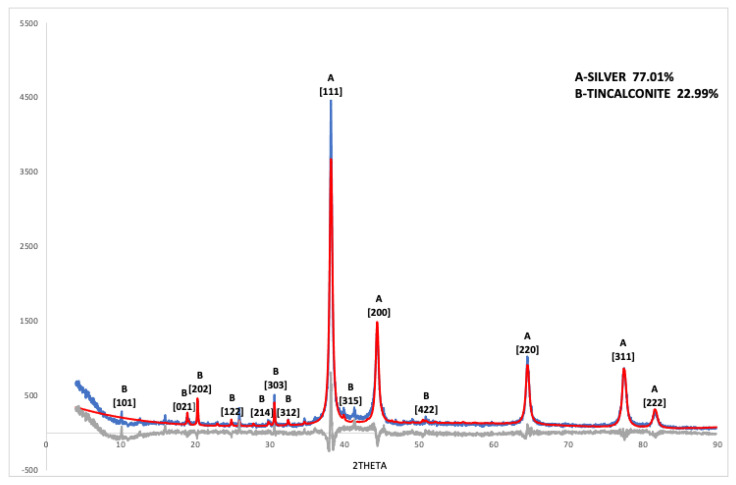
XRD measurements of powders obtained by precipitation from the aqueous dispersion (blue) and Rietveld refinement (red). Difference (Measured pattern—Rietveld refinement) (grey).

To obtain detailed information about the composition, crystalline structure, and mean size of nanocrystalline silver particles in the nanoparticle dispersion used to prepare the modified membranes, a quantitative phase analysis of the diffraction pattern of the precipitated particles (Figure 1) was performed using the computer program TOPAS 4.2. Precipitation of the particles was carried out by taking 1 mL of the nanoparticle dispersion, adding 1 mL of acetone, and leaving the mixture for 1 min in an ultrasound bath. After centrifugation, the supernatant was removed, and the nanoparticles were mixed thoroughly in 1 mL of water with the aid of the ultrasonicator to ensure dispersion. After that, 1 mL of acetone was added, and the entire washing procedure was repeated 6 times. The particles were dried under vacuum for several days.

For the refinement, a crystal structure of silver according to PDF 01-087-0717 Silver 3C was applied. The atomic positions were determined by Rietveld refinement, obtaining a unit cell value of a = 4.08681(37) Å assuming Fm-3m symmetry and an average silver particle size of 22.6 nm (Table 3). For the fit of the sample, a Rwp value of 20.82% was obtained, Rp: 15.82, and GOF: 2.89. In addition to silver (77%), the sample contained 23% of tincalconite (PDF 00-007-0277, Na_2_B_4_O_7_.5H_2_O syn), resulting from precipitation during the washing procedure of the oxidation products of the borohydride ions. As demonstrated from XPS characterization, boron/sodium compounds were not present in the modified membranes, showing that the soluble products of silver reduction do not affect the membrane composition during preparation.

### 3.3. XPS Results

#### 3.3.1. Silver Nanoparticles Dispersion

**Figure 2:**
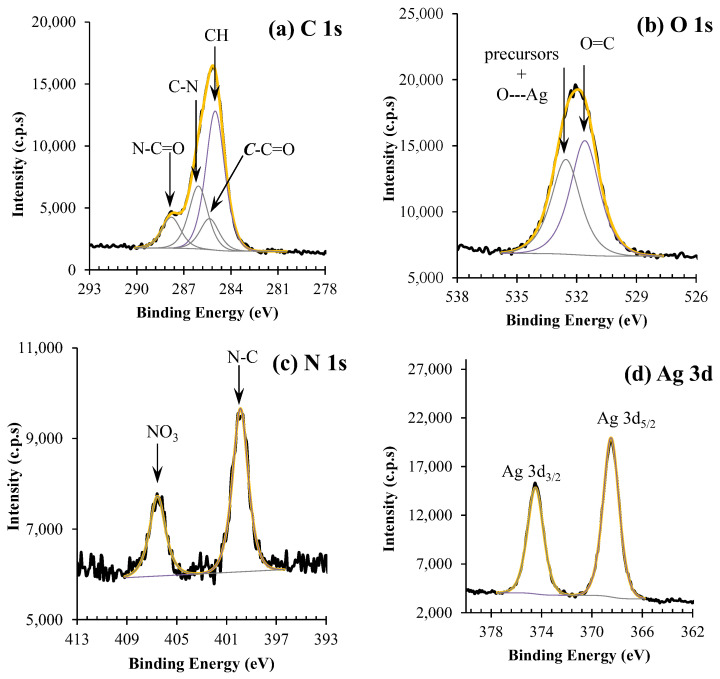
XPS regions of (**a**–**d**) for silver nanoparticle dispersions (AgNP).

**Figure 3:**
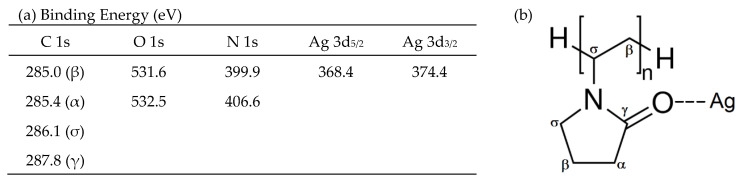
(**a**) Binding energy values for the PVP-stabilizing silver nanoparticle dispersion (AgNP), (**b**) Chemical structure of PVP and possible coordination with silver.

XPS detailed regions of silver nanoparticles dispersion (AgNP) and qualitative XPS analysis are shown in Figure 2 and Figure 3.

Four peaks centered at 285.0, 285.4, 286.1, and 287.8 eV, typical of PVP, were fitted into the C 1s envelope. The detailed assignment is presented in Figure 3.

The O 1s region (Figure 2b) was fitted with two peaks: one centered at 531.6 eV, which is attributed to oxygen double bound to carbon, as the one found in PVP; and another one, centered at 532.5 eV, assigned to oxygen from nitrate groups, which still remains in the colloidal system, to the interaction between oxygen and silver nanoparticles, and to some retained water [15]. The N 1s spectrum (Figure 2c) presents two peaks located at 399.9 and 406.6 eV, attributed to the N atom in PVP and NO_3_^−^ (from precursor silver nitrate), respectively. The Ag 3d region of silver nanoparticles (Figure 2d) is a simple doublet with an energy separation of 6.0 eV. The Ag 3d_5/2_ component is centered at 368.4 eV and, regarding the dispersion of the values reported in the literature, can be assigned to any oxidation state. However, the oxidation state can be identified from the Auger parameter, which is the sum of the experimental binding energy of the photoelectron peak Ag 3d_5/2_ and the experimental kinetic energy of the two most intense peaks of the Ag MNN Auger structure [16]. In this case, the computed value of 718.2 eV suggests that silver is oxidized or that the silver nanoparticles are covered with a layer of oxidized silver [16].

#### 3.3.2. CA and CA/Ag Membranes

**Figure 4:**
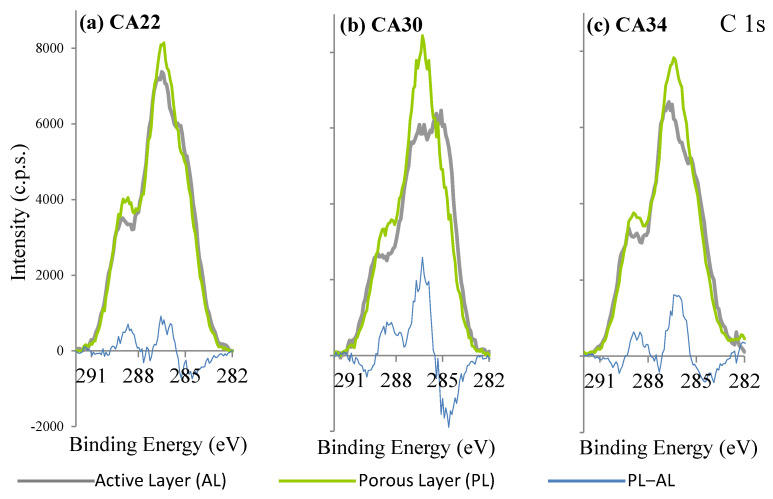
XPS C 1s regions of active layers (AL) and porous layers (PL) of CA membranes. The blue line is the difference between the PL and AL spectra.

**Figure 5:**
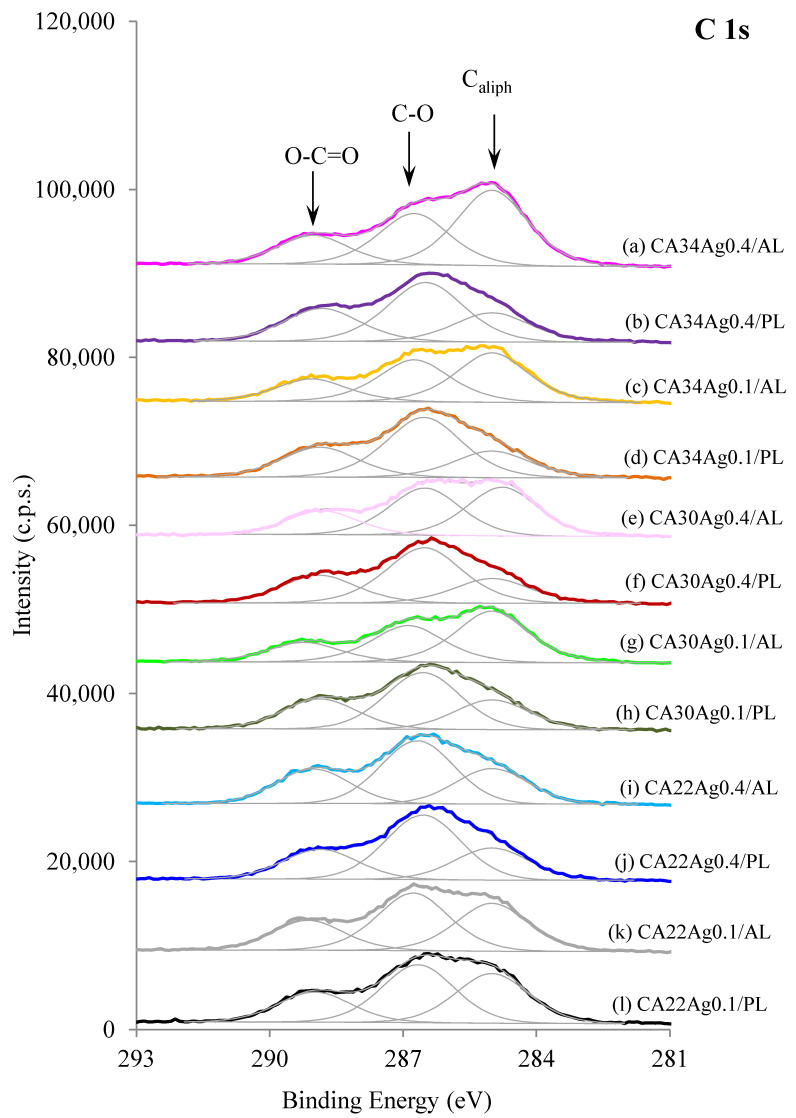
XPS C 1s regions of active layers (AL) and porous layers (PL) of CA/Ag membranes with 0.1% wt Ag and 0.4% wt Ag. A constant was added to each spectrum (except for l) for clarity.

The results for CA and CA/Ag membranes XPS C 1s regions are displayed in Figure 4 and Figure 5, respectively. The C 1s regions, for all the membranes, were fitted with three peaks centered at 285.0 eV (binding energy used to correct the charge shifts), attributed to aliphatic carbons, and at 286.7 ± 0.1 eV and 289.0 ± 0.2 eV, attributed to carbons from CA: C-O and carbons of ester groups, respectively [17]. Another peak at ~288 eV, assignable to carbon in the cellulose ring bonded to two oxygen atoms, exists in cellulose-derived polymers. However, given its low intensity (1/5 of C-O peak intensity), there was no need to add it in the fitting.

The C 1s regions of the three silver-free membranes (Figure 4a–c), show some differences between the dense layer, also designated as the active layer (AL), and the porous (PL) layer. The membrane with larger difference between AL and PL is the CA30, and the one with smaller differences is the CA22, which is also the one with the smallest hydraulic permeability. The relative amount of aliphatic carbon atoms for each membrane is larger in AL than in the PL spectra. This fact may be associated with the presence of acetone residues in AL, which are expected to be larger in the denser layer (AL).

Little difference between AL and PL was observed for the silver-free CA22 membrane and for the CA22 silver-containing membranes (CA22Ag0.1 and CA22Ag0.4, Figure 5i–l), having very similar C 1s regions for AL and PL surfaces. In the other membranes, with higher hydraulic permeabilities, different C 1s regions are observed (Figure 5a–h): the relative intensity of the peak attributed to aliphatic carbon atoms (285 eV) is larger in AL than in PL, as observed for silver-free membranes.

**Figure 6:**
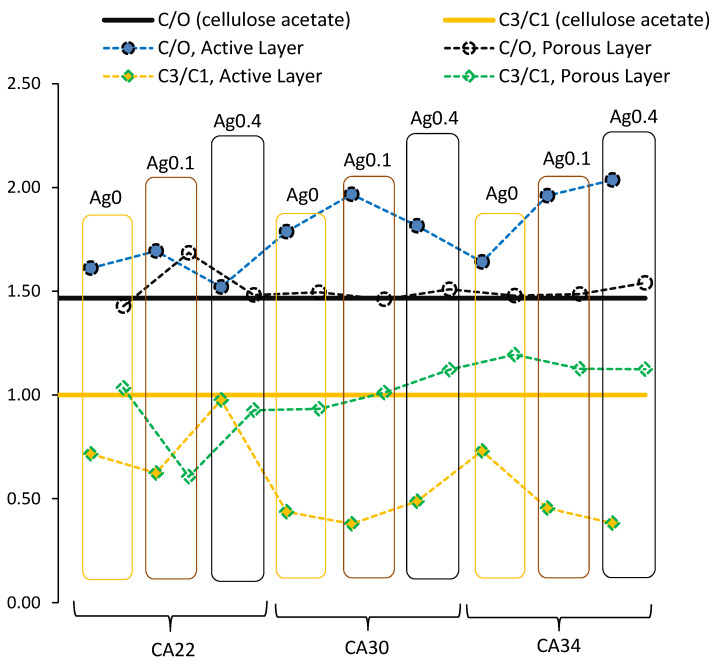
Atomic ratios C/O and C3/C1. The horizontal lines at 1.47 and 1 correspond, respectively, to stoichiometric ratios C/O and C3/C1 in cellulose acetate used to prepare the membranes.

The atomic ratios C/O and C3/C1 were computed from the C 1s and O 1s (not shown) regions, where C and O were computed from the total areas of C 1s and O 1s divided by the respective sensitivity factors, C3 is the amount of carbon atoms in O–C=O groups (peak at 289 eV), and C1 is the amount of aliphatic carbons (peak at 285 eV). These ratios can give a clear insight into the differences between AL and PL of CA and CA/Ag membranes. The results are gathered in Figure 6.

For the cellulose acetate here used, the nominal ratio C/O is 1.47, and the ratio C3/C1 is 1. These stoichiometric values are represented as black and yellow horizontal lines respectively, in Figure 6. The full symbols are the experimental ratios for the membranes AL, and the empty symbols are the experimental ratios for the membranes PL. The results show, very clearly, that membrane porous layers have a composition closer to the one found in cellulose acetate than do the active layers. In fact, active layers seem to be richer in carbon than are the porous layers, particularly in aliphatic carbon, as noticed above in Figure 5a–h. Such difference may be due to the larger retention of some of the solvents used in the casting solution by the polymer-denser active layer. The exceptions are the membranes of lowest hydraulic permeabilities with 0.1 and 0.4 % wt Ag (CA22Ag0.1 and CA22Ag0.4, respectively), which present comparable C/O and C3/C1 ratios for both layers. This is in agreement with the results obtained for the C 1s region presented in Figure 5i–l.

**Figure 7:**
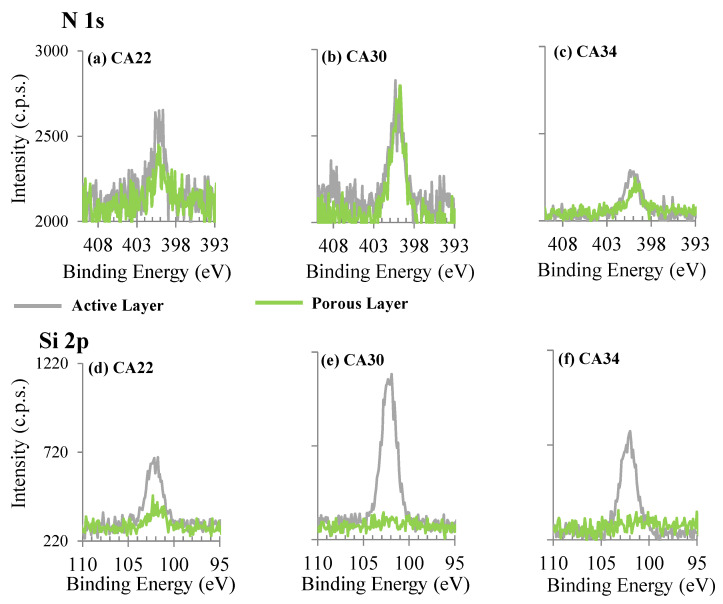
XPS N 1s and Si 2p regions of active layers (AL, grey spectra) and porous layers (PL, green spectra) of CA membranes (without Ag).

The regions of N 1s and Si 2p were also investigated for the CA membranes (Figure 7). The presence of nitrogen in the CA membranes (silver-free membranes) was not expected at all. However, N 1s is clearly detected at the surface of active and porous layers of membranes without PVP stabilized AgNPs, as shown in Figure 7. The presence of nitrogen at the membrane surfaces may be attributed to the formamide used in the formulation of the casting solution, which could have been retained on the membrane during the phase inversion process, not diffusing completely into the aqueous phase. Silicon is clearly accumulating at the active layer surface. Silicon presence results most likely from contamination carried by the commercial CA polymer and may be washed from the porous layer by the coagulation bath.

The source of nitrogen in CA/Ag membranes, in addition to the possible contribution of formamide used in casting solutions, can also have the input of the PVP used to stabilize the silver nanoparticles, avoiding agglomeration. High surface energy of the ultrasmall particles is normally controlled to overcome aggregation and loss of properties; PVP capability to protect the particles, and yield stable dispersions, has been previously reported [8,9,18,19]. Silicon is also detected in CA/Ag membranes. Its relative amount follows what is observed in the absence of Ag, being in a much larger amount at the AL surface than at the PL surface.

**Figure 8:**
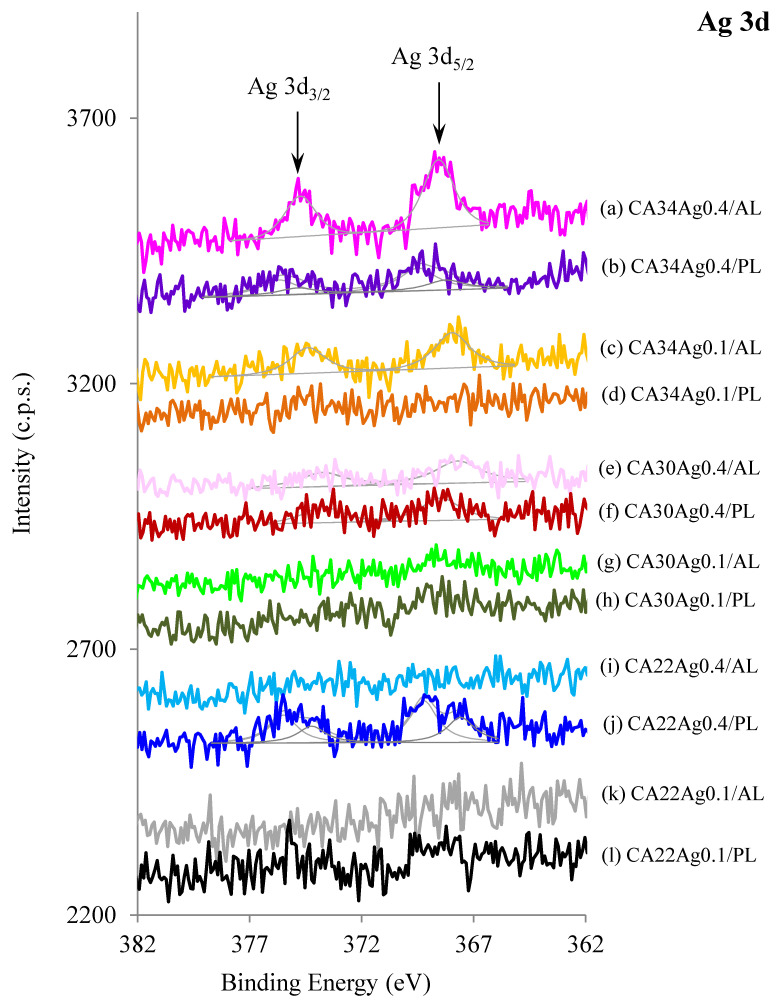
XPS Ag 3d regions of active layers (AL) and porous layers (PL) of CA/Ag with 0.1% wt Ag and 0.4% wt Ag membranes.

The Ag 3d XPS regions for CA/Ag membranes are displayed in Figure 8. They are doublets with energy separation of 6.0 eV. For the porous layers, two doublets were needed to fit the Ag 3d profile. This is, in principle, due to differential charge effects which are expected to occur in very rough surfaces. In some of the CA/Ag membranes, silver is not detected, which may not mean that nanoparticles are absent but that the silver nanoparticles may be located beyond the maximum depth probed by XPS, i.e., “buried” at more than 10 nm from the surface.

Membranes prepared with higher silver content (0.4% wt) are those where silver is more easily detected, particularly in the active layer of the membrane CA34. This corresponds well with the observation made by Figueiredo et al. (2015) [8] that most of the particles, particularly the smaller ones, concentrate in the active layers. In fact, for these membranes (CA34Ag0.4), a decrease was observed in the hydraulic permeability when compared with the CA34Ag0.1, which is associated with the pore-blocking caused by the AgNP in the AL. For the other two tighter membranes, CA30 and CA22, the respective increase of the membrane matrix density makes difficult the detection of AgNP leading to the decrease of the Ag 3d XPS signal of AL. On the other hand, it was observed that a decrease in silver content (0.4 to 0.1% wt) also leads to an expected decrease of the Ag 3d XPS signal of AL.

The Ag 3d XPS signals observed for the porous layers of the CA30 and CA34 are less intense than the ones for AL due to the preferential presence of AgNP in the AL [8]. In the CA22 membranes, there is a measurable Ag 3d signal from the porous layer, which is not observed for the active layer. This is because the active layers of CA22 membranes present a very dense matrix.

To identify the oxidation state of silver, one cannot rely only on the Ag 3d_5/2_ binding energy; instead, the Auger parameter (AP) must be computed [16]. However, the calculation of the Auger parameter was not possible for the CA/Ag membranes because the Auger structure (Ag MNN), needed to compute this parameter, was not detected. This effect can occur when Auger electrons (which have a kinetic energy lower than the Ag 3d photoelectrons) are coming from buried silver nanoparticles and are, then, attenuated by the membrane. Nevertheless, the Auger parameter found for the silver nanoparticle suspension (AP = 718.2 eV), the same suspension which was characterized by XRD, as mentioned above, suggests that silver is oxidized or that silver nanoparticles are covered with a layer of ionized silver. Since XRD results show that silver nanoparticles present a typical metallic silver structure, one can assume the surface of the particles is either oxidized or the silver is chemically interacting with the PVP molecules adsorbed. This is an important result, as the slow release of ionized silver, as a result of the equilibrium 2Ag_2_O + 4H^+^ → 4Ag^+^ + 2H_2_O, favors the continuous Ag^+^ release from the nanoparticles surface, particularly in aerobic media as the one used in this work, where the oxidation reaction 4Ag(0) + O_2_ → 2Ag_2_O leads to a slow but continuous supply of silver ions [20,21,22,23,24]. In this sense, XPS and XRD results complement each other.

### 3.4. Membranes Bactericide Properties

To investigate the bactericide properties of the CA/Ag membranes, specific tests were performed against *E. coli*. The general bacteria growth in water for a long period (80 days) was also analyzed.

#### 3.4.1. Surface Test

**Figure 9:**
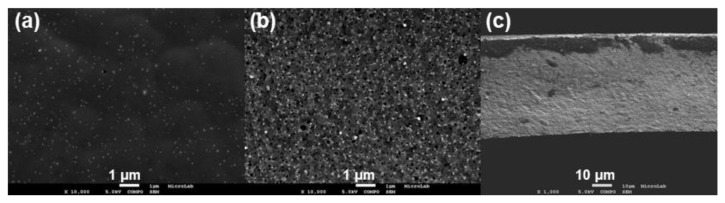
FESEM images of (**a**) active layer, (**b**) porous layer, and (**c**) cross-section of CA30Ag0.1 membrane (adapted from Ref. [8]. 2014, John Wiley and Sons).

The surface tests were described previously in Section 2.7.1 and were conducted for the three membrane structures (CA22, CA30, and CA34) with different AgNP contents (0%, 0.1%, and 0.4%). As can be seen from Figure 9 (adapted from [8]), the active and porous layers of membranes prepared under the same experimental conditions present very different characteristics; therefore, the bacterial growth inhibition pattern of the membrane layers was evaluated to have some insights about the possibility for these membranes preventing the biofouling phenomena. Before the tests, all the membranes were sterilized in a UV camera for 30 min on each side. No bacterial growth was observed following sterilization.

**Figure 10:**
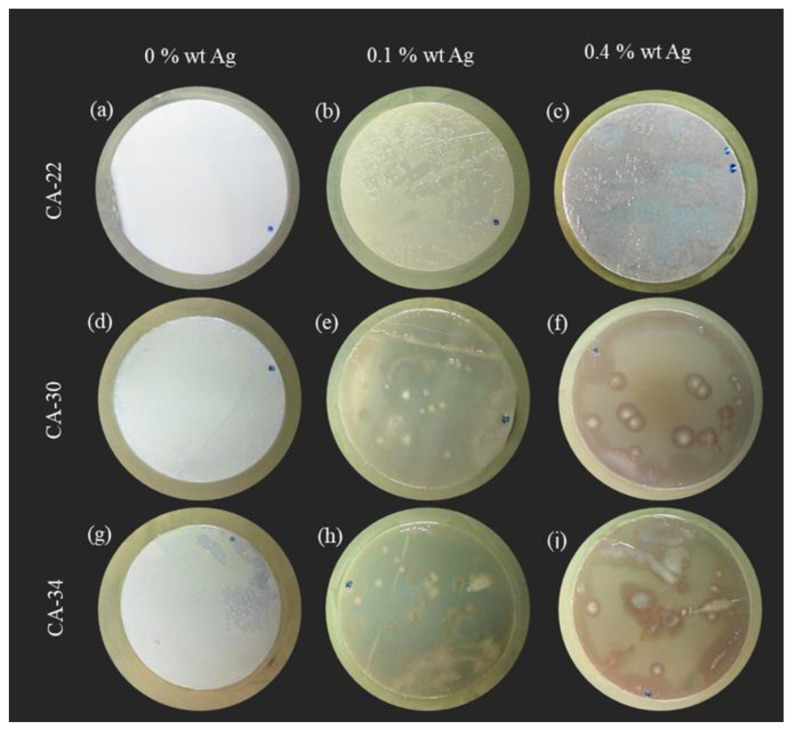
Results of *E. coli* growth inhibition tests on the active layers of CA membranes (**a**) CA22, (**d**) CA30, and (**g**) CA34; CA/Ag membranes with 0.1wt% Ag (**b**) CA22Ag0.1, (**e**) CA30Ag0.1, and (**h**) CA34Ag0.1; and CA/Ag membranes with 0.4wt% Ag (**c**) CA22Ag0.4, (**f**) CA30Ag0.4, and (**i**) CA34Ag0.4.

From the results presented in Figure 10, it was possible to observe, for the silver-free membranes (Figure 10a,d,g), a generalized growth of *E. coli* in all the membrane active layer surfaces, forming a bacteria film on the membrane surface, visible even to the naked eye.

For the membranes with AgNP, the results obtained with the CA22Ag0.1 and CA22Ag0.4 (Figure 10b,c) show different zones on the membrane active layer, indicating that the *E. coli* growth was not generalized, with existing parts of the membrane surface exhibiting *E. coli* growth inhibition. In the surface test conducted for the active layer of membrane structures CA30 and CA34 with AgNP (0.1% and 0.4%), evident growth inhibition was observed, as only a few *E. coli* colonies (white dots) were detected in the Figure 10e,f,h,i.

**Figure 11:**
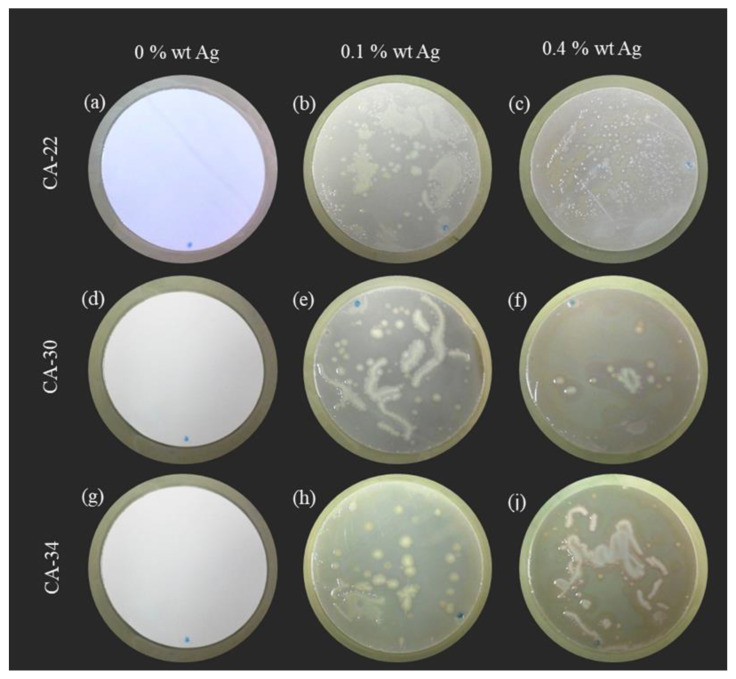
Results of *E. coli* growth inhibition on the porous layer of CA membranes (**a**) CA22, (**d**) CA30, and (**g**) CA34; CA/Ag membranes with 0.1wt% Ag (**b**) CA22Ag0.1, (**e**) CA30Ag0.1, and (**h**) CA34Ag0.1; and CA/Ag membranes with 0.4wt% Ag (**c**) CA22Ag0.4, (**f**) CA30Ag0.4, and (**i**) CA34Ag0.4.

The results obtained from the surface tests of the membrane porous layers are presented in Figure 11. The results obtained from the silver-free membranes porous layer indicate that *E. coli* growth inhibition was not observed (Figure 11a,d,g).

For the membranes CA22 with AgNP (0.1 and 0.4%), a slight increase in the growth inhibition for the membrane porous layer was observed, compared with the results obtained for the active layers of the CA22Ag0.1 and CA22Ag0.4 membranes. This is in line with the XPS results, which revealed a higher Ag concentration in the porous layer. For the CA30 and CA34 with AgNP, the results for the porous layer (Figure 11e,f,h,i) are comparable to those obtained for the active layers (Figure 10e,f,h,i), where significant *E. coli* growth inhibition is observed.

The difference observed in growth inhibition among the silver-containing membranes CA22 (CA22Ag0.1 and CA22Ag0.4), CA30 (CA30Ag0.1 and CA30Ag0.4), and CA34 (CA34Ag0.1 and CA34Ag0.4) may be related to the fact that the structure of the CA22 membranes is much tighter than the one of the CA30 and CA34 membranes. The presence of AgNP in the proximity of the active layer surfaces is also more difficult to detect for the CA22 silver-containing membranes (Figure 8). The differences between the surfaces of the active and porous layers of CA22Ag0.1 and CA22Ag0.4 membranes are consistent with the fact that a more porous structure promotes a better interaction between *E. coli* and the AgNP and, therefore, leads to high growth inhibition for the porous layers of CA22 silver-containing membranes. In addition, the XPS reveals that the porous layer contains a larger relative amount of silver.

As previously presented (Figure 8) and discussed, the XPS results indicate that in some membrane samples, the silver presence was not detected, which may be due to the fact that the AgNP might be located more than 10 nm away from the membrane’s outermost surface. In addition to the difficulty of detecting the silver signal by XPS for the CA22 silver-containing membranes, the silver signal was perceptible for the porous layer of CA22Ag0.1 membrane (although not quantified due to the very poor signal-to-noise ratio), which is consistent with the lowest *E. coli* growth inhibition results obtained for the surface test of CA22 membranes.

#### 3.4.2. Suspension Test

To complement the results obtained in the surface test, suspension tests were performed by immersing the membrane samples in *E. coli*-inoculated suspensions at room temperature (20 °C), different from the temperature used in the surface tests, which was considered ideal for the *E. coli* growth (37 °C).

**Figure 12:**
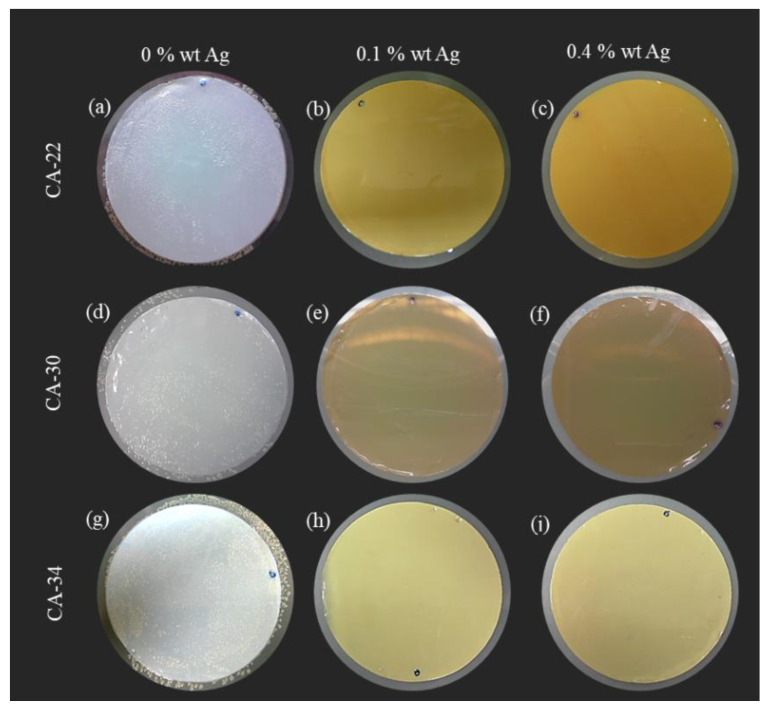
Results of *E. coli* growth inhibition on the membrane for the suspension test of CA membranes (**a**) CA22, (**d**) CA30, and (**g**) CA34; CA/Ag membranes with 0.1wt% Ag (**b**) CA22Ag0.1, (**e**) CA30Ag0.1, and (**h**) CA34Ag0.1; and CA/Ag membranes with 0.4wt% Ag (**c**) CA22Ag0.4, (**f**) CA30Ag0.4, and (**i**) CA34Ag0.4.

**Figure 13:**
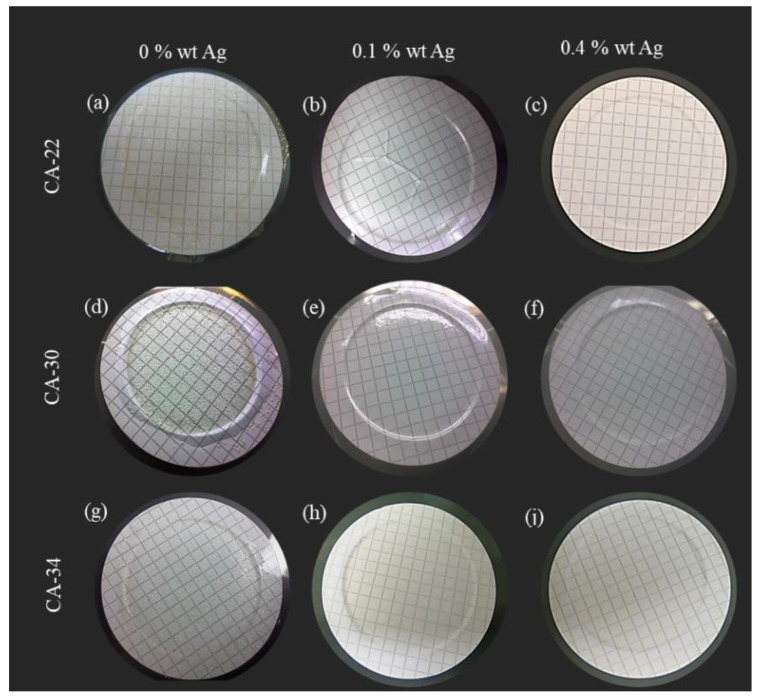
Results of *E. coli* growth inhibition on the suspension for the suspension test of CA and CA/Ag membranes. Silver-free membranes: (**a**) CA22, (**d**) CA30, and (**g**) CA34; CA/Ag membranes with 0.1wt% Ag: (**b**) CA22Ag0.1, (**e**) CA30Ag0.1, and (**h**) CA34Ag0.1; CA/Ag membranes with 0.4wt% Ag: (**c**) CA22Ag0.4, (**f**) CA30Ag0.4, and (**i**) CA34Ag0.4.

The membranes and the suspensions were evaluated in terms of *E. coli* growth inhibition, according to the experiments previously described in the Materials and Methods section. Figure 12 presents the membrane samples after immersion in the inoculated suspension (24 h) and incubation in a Petri dish with YEA at 37 °C (24 h), and Figure 13 presents the incubated filters used to filter de-inoculated suspension.

From the results presented in Figure 12, it can be seen very clearly that there is a total *E. coli* growth inhibition for all the membrane structures containing AgNP (Figure 12b,c,e,f,h,i). In contrast, the silver-free membranes (Figure 12a,d,g) show no *E. coli* growth inhibition.

Figure 13 presents the results for the membrane filters used to filter the *E. coli* suspensions, where the membranes were immersed for the suspension test (Figure 12). For the suspensions in contact with silver-free membranes, full development of bacterial growth was verified in the membrane filters (Figure 13a,d,g). On the other hand, the suspensions in contact with CA/Ag membranes lead to a total absence of *E. coli* colonies in the membrane filters (Figure 13b,c,e,f,h,i).

In the suspension test, with experimental conditions which are similar to the ones of the water filtration processes, there is clear evidence that the CA/Ag membranes promote total growth inhibition independently of the membrane structure and the silver content.

#### 3.4.3. Cell Death Test

**Figure 14:**
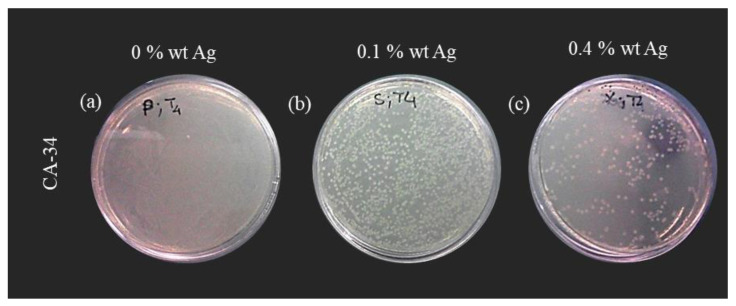
*E. coli* colonies after the incubation of the suspensions contacting with the membranes (1093 min). (**a**) Silver-free membranes (CA34); (**b**) CA/Ag membranes with 0.1wt% (CA34Ag0.1); and (**c**) CA/Ag membranes with 0.4wt% Ag (CA34Ag0.4).

The previous results (Section 3.4.1 and Section 3.4.2) demonstrate that the presence of silver nanoparticles introduces an antibacterial effect in the CA/Ag membranes, independent of the different silver content. To clarify the silver nanoparticles content effect on the antibacterial properties of silver-containing membranes, the cell death was performed for the CA34 membranes series because the membranes CA34Ag0.1 and CA34Ag0.4 are those where silver is more easily detected by XPS results. The membranes CA34, CA34Ag0.1, and CA34Ag0.4 were tested at 37 °C. The results are presented in Figure 13 and Figure 14.

The cell death test was carried out for 18 h and samples of the suspension were collected during this period. Figure 14 presents the image of the Petri dishes incubated with the sample collected at 1093 min of membrane/*E. coli* suspension contact. The results show a generalized growth of *E. coli* for the suspension in contact with silver-free membranes (Figure 14a). In contrast, there is growth inhibition for the tests conducted with the CA34Ag0.1 and CA34Ag0.4 membranes, resulting in a decrease in the number of *E. coli* colonies, which is even more pronounced for the CA34Ag0.4 membrane (Figure 14c).

**Figure 15:**
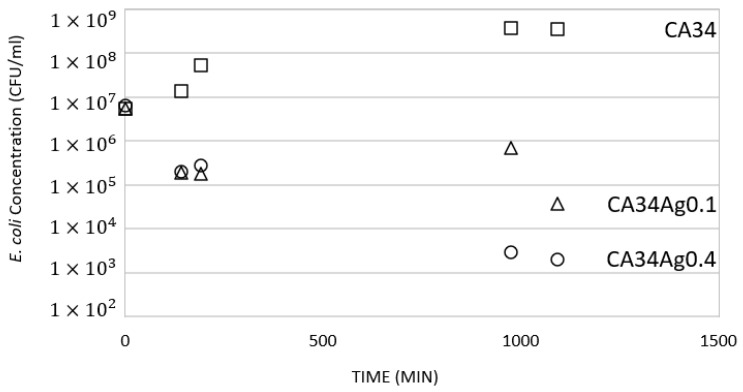
Variation of *E. coli* concentration (CFU/mL) at different contact times (143 min, 190 min, 975 min and 1093 min) of the suspensions with the membranes (□, CA34; △, CA34Ag0.1; and ⚬, CA34Ag0.4).

Figure 15 displays, for the CA34, CA34Ag0.1, and CA34Ag0.4 membranes, the variation of *E. coli* concentration (CFU/mL) at different contact times of the suspensions with the membranes.

The concentration of *E. coli* decreases with the time of contact between the suspension and the CA/Ag membranes (CA34Ag0.1 and CA34Ag0.4). On the contrary, the concentration of *E. coli* increases with the time of contact between silver-free membranes and the *E. coli* suspension. For CA nanofiltration membranes incorporating silver nanoparticles or silver ion-exchanged β-zeolite, Beisl et al. (2019) [7] also verified a decrease in the concentration of *E. coli* along the time of contact between the suspension and the membranes.

#### 3.4.4. Growth Inhibition of Bacteria in the Water

**Figure 16:**
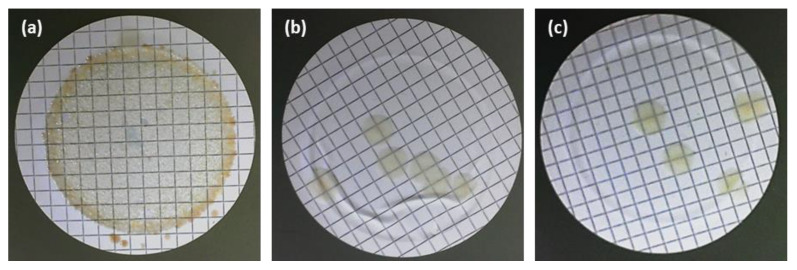
Inhibition growth test of microorganisms in water with membranes. (**a**) Silver-free membranes (CA30); (**b**) CA/Ag membranes with 0.1wt% Ag (CA30Ag0.1); and (**c**) CA/Ag membranes with 0.4wt% Ag (CA30Ag0.4).

To consolidate the capability of silver-containing membranes to inhibit the growth of bacteria in water, the membranes CA30, CA30Ag0.1, and CA30Ag0.4 were immersed in water for a long period (80 days). The results are presented in Figure 16.

The silver-free membranes showed generalized bacterial growth (Figure 16a). The silver-containing membranes, CA30Ag0.1 and CA30Ag0.4, display a strong reduction in the number of colonies in the filters. The CA30Ag0.1 membrane (Figure 16b) showed six colonies, while the CA30Ag0.4 membrane (Figure 16c) presented four colonies. These results provide strong evidence of bacterial growth inhibition in water contacting CA/Ag membranes, independently of structure, silver content, aggregation degree, and distribution of the particles in both dense and porous layers.

## 4. Conclusions

In this work, cellulose acetate/silver nanoparticles integral asymmetric ultrafiltration membranes were synthesized, characterized, and tested for their bactericidal properties. Combined XRD and XPS characterization showed that the silver nanoparticles are covered with ionic silver, able to solubilize and exert bactericide activity.

The active and porous layers’ surfaces display different compositions, with the exception of the less permeable membranes, CA22Ag0.1 and CA22Ag0.4, that present similar C/O and O–C=O/(C-C, C-H) ratios in both surfaces. The porous layers composition of the more permeable membranes (CA30, CA30Ag0.1, CA30Ag0.4, CA34, CA34Ag0.1, and CA34Ag0.4) is closer to the composition of the CA polymer.

The silver-free membranes present a generalized growth of *E. coli*. This is in contrast with the inhibition pattern displayed by the membranes with AgNP. The antibacterial tests carried on the membrane surfaces of the less permeable membranes, CA22Ag0.1 and CA22Ag0.4, evidence a lower *E. coli* growth inhibition when compared with the more permeable membranes (CA30, CA30Ag0.1, CA30Ag0.4, CA34, CA34Ag0.1, and CA34Ag0.4) that present almost complete growth inhibition. Furthermore, the porous layers of the CA22Ag0.1 and CA22Ag0.4 show a slight increase in the *E. coli* growth inhibition in comparison with the corresponding active layers. This is in line with the XPS results which revealed a higher Ag concentration in the porous layer. For the more permeable membranes (CA30, CA30Ag0.1, CA30Ag0.4, CA34, CA34Ag0.1, and CA34Ag0.4), the significant *E. coli* growth inhibition is comparable in both active and porous layers. For those membranes, XPS reveals a higher amount of silver in the active layer than in the porous one, as already stated in [8]. However, the access to the AgNP in the porous layer is higher. Apparently, the two effects compensate for each other leading to similar bactericide properties in both layers.

## Figures and Tables

**Table 1:** Casting solutions compositions and film casting conditions of the CA membranes free of silver (CA22, CA30, and CA34), with 0.1wt% Ag (CA22Ag0.1, CA30Ag0.1, and CA34Ag0.1) and with 0.4wt% Ag (CA22Ag0.4, CA30Ag0.4, and CA34Ag0.4).

Casting Solution (wt%)									
Membrane	CA22	CA22Ag0.1	CA22Ag0.4	CA30	CA30Ag0.1	CA30Ag0.4	CA34	CA34Ag0.1	CA34Ag0.4
Cellulose acetate	17.0	16.4	15.3	17.0	16.4	15.3	17.0	16.4	15.3
Formamide	22.0	21.2	19.8	30.0	29.0	27.0	34.0	32.8	30.6
Acetone	61.0	58.9	54.9	53.0	51.1	47.7	49.0	47.3	44.1
AgNPs									
Dispersion	-	3.32	9.59	-	3.32	9.59	-	3.32	9.59
Silver	-	0.14	0.41	-	0.14	0.42	-	0.14	0.41
**Casting Conditions**									
Temperature of solution (°C)					20–25				
Temperature of atmosphere (°C)					20–25				
Solvent evaporation time (min)					0.5				
Gelation medium					Water at temperature of 0–3 °C during 1–2 h				

**Table 2:** CA and CA/Ag pure water fluxes (Jp), hydraulic permeabilities (Lp), and MWCO (membrane surface area: 13.2 × 10^−4^ m^2^).

Membrane	Jp (kg m^−2^ h^−1^)			Lp (kg m^−2^ h^−1^ bar^−1^)	MWCO (kDa)
	1 bar	2 bar	3 bar		
CA22	2.62	6.62	11.03	3.50	4.17
CA22Ag0.1	5.31	13.90	21.87	7.05	6.86
CA22Ag0.4	9.75	22.40	33.89	11.16	15.35
CA30	24.43	62.73	99.59	32.05	8.32
CA30Ag0.1	55.06	136.59	202.57	66.85	17.58
CA30Ag0.4	58.28	123.51	176.93	59.72	26.52
CA34	68.55	176.68	236.81	80.88	31.43
CA34Ag0.1	76.44	187.99	243.42	84.48	41.05
CA34Ag0.4	62.60	123.17	172.80	59.10	31.96

**Table 3:** Rietveld refinement of silver nanoparticles.

Phase Name:			Silver		Scale:		0.0007801(63)
R-Bragg:			2.028		Cell Mass:		431.470
Space Group:			Fm-3m		Cell Volume (Å^3^):		68.258(19)
Wt%—Rietveld:			77.0(12)				
**Crystallite Size**							
Cryst. Size Lorentzian (nm):			22.26(28)				
Cryst. Linear Absorp. Coeff. (1/cm):			2247.09(61)				
Crystal Density (g/cm^3^):			10.4965(29)				
Preferred Orientation (Dir 1: 1 1 1):			0.7610(77)				
**Lattice parameters**							
a (≈):			4.08681(37)				
Site	NP	X	Y	Z	Atom	Occ.	Beq
Ag	4	0.00000	0.00000	0.00000	Ag	1	0

## Data Availability

Not applicable.
